# A Prediction Model for Contralateral Central Neck Lymph Node Metastases in Unilateral Papillary Thyroid Cancer

**DOI:** 10.1155/2021/6621067

**Published:** 2021-06-30

**Authors:** Hai-Long Tan, Bo-Qiang Huang, Gui-You Li, Bo Wei, Pei Chen, Hui-Yu Hu, Mian Liu, Deng-Jie Ou-Yang, Qiong Yang, Zi-En Qin, Qi-Man Shi, Ning Li, Peng Huang, Shi Chang

**Affiliations:** ^1^Department of General Surgery, Xiangya Hospital Central South University, Changsha 410008, Hunan, China; ^2^National Clinical Research Center for Geriatric Disorders, Xiangya Hospital Central South University, Changsha 410008, Hunan, China; ^3^Clinical Research Center For Thyroid Disease In Hunan Province, Changsha, Hunan, China

## Abstract

The health problems caused by the frequent relapse of papillary thyroid carcinoma (PTC) remain a worldwide concern since the morbidity rate of PTC ranks the highest among thyroid cancers. Residues from contralateral central lymph node metastases (con-CLNM) are the key reason for persistence or recurrence of unilateral papillary thyroid carcinoma (uni-PTC); however, the ability to assess the status of con-CLNM in uni-PTC patients is limited. To clarify the risk factors of con-CLNM, a total of 250 patients with uni-PTC who underwent total thyroidectomy and bilateral central lymph node dissection were recruited in this study. We compared the clinical, sonographic, and pathological characteristics of patients with con-CLNM to those without con-CLNM and established a nomogram for con-CLNM in uni-PTC. We found that male sex, without Hashimoto's thyroiditis, present capsular invasion, with ipsilateral lateral lymph node metastases, and the ratio of ipsilateral central lymph node metastases ≥0.16 were independent con-CLNM predictors of uni-PTC (ORs: 2.797, 0.430, 2.538, 2.202, and 26.588; 95% CIs: 1.182–6.617, 0.211–0.876, 1.223–5.267, 1.064–4.557, and 7.596–93.069, respectively). Additionally, a preoperative nomogram for the prediction of con-CLNM based on these risk factors showed good discrimination (C-index 0.881; 95% CI: 0.840–0.923; sensitivity 85.3%; specificity 76.0%) and good agreement via the calibration plot. Our study provided a way to quantitatively and accurately predict whether con-CLNM occurred in patients with uni-PTC, which may guide surgeons to evaluate the nodal status and perform tailored therapeutic central lymph node dissection.

## 1. Introduction

Papillary thyroid carcinoma (PTC) is the most common pathological type of thyroid cancer, with an incidence increasing dramatically worldwide [1–3]. Since malignancy is less severe after surgery and postoperative adjuvant therapy, most PTC patients have an excellent prognosis and long-term survival [4, 5]. However, cervical lymph node metastasis (LNM), which occurs in approximately 20–90% of PTC patients, makes regional recurrence an important issue [6, 7]. Moreover, central lymph node metastasis (CLNM) is an important prevalent factor affecting cancer recurrence in PTC patients [8].

A stepwise fashion has been widely accepted for cervical lymph node metastasis of PTC, which means primary tumor cells of the PTC initially spread from the thyroid gland to the ipsilateral central compartment (level VI) and subsequently to the lateral compartment on the same side (level II, III, IV, and V) followed by the contralateral central compartment, lateral compartment, and mediastinal lymph nodes [9–12]; therefore, when cervical lymph node metastases are detected (cN1) by either clinical or radiological means, ipsilateral central lymph node dissection (CLND) in unilateral papillary thyroid carcinoma (uni-PTC) is well accepted [13, 14]. Besides, approximately 20%–25% uni-PTC with pathological positive ipsilateral CLNM (ipsi-CLNM) were found occurring contralateral CLNM (con-CLNM) [15, 16], especially in those patients with primary tumor size >1 cm [15, 17]. In addition, 13.2%–23.0% of residual nodal metastases occur in the contralateral central neck compartment in uni-PTC patients who underwent ipsilateral CLND, which was a significantly higher risk, compared with those who underwent bilateral CLND [18, 19]. Therefore, therapeutic ipsilateral or bilateral central lymph node dissection is recommended for patients with clinically positive lymph nodes [20–22]. However, it has been controversial whether to perform contralateral prophylactic CLND in uni-PTC patients with clinically negative lymph nodes in the contralateral neck central compartment since contralateral prophylactic CLND is considered to increase the risk of morbidities such as recurrent laryngeal nerve injury and hypoparathyroidism [23]. The current dilemma is how to weigh minimization of the morbidity of local recurrence and reoperation against potential increased accidental injury and complications. Therefore, an accurate preoperative evaluation of con-CLNM helps in determining the range of surgery and improving the tumor-free survival and quality of life of uni-PTC patients.

It is crucial for surgeons to preoperatively evaluate the status of lymph node in the contralateral central neck compartment when determining the extent of lymph node dissection (LND) for uni-PTC patients. However, few studies on defining the predictors of con-CLNM for uni-PTC patients have been reported. In this study, a logistic regression model was used to analyze the clinicopathological features of con-CLNM for uni-PTC to develop a nomogram for predicting con-CLNM, which helped in determining the surgical range, further decreasing the risk of residual positive lymph nodes in the contralateral central compartment, and reducing recurrence in uni-PTC patients.

## 2. Materials and Methods

### 2.1. Patients and Study Design

This study received ethical approval from the Ethics Committee of Xiangya Hospital Central South University, and all patients consented to the anonymous use of their data for research prior to surgery. Between January 2016 and October 2019, 5268 PTC patients underwent thyroidectomy at the Department of General Surgery, Xiangya Hospital Central South University. Patients with any of the following conditions were excluded: clinicopathologic data incomplete, bilateral or isthmus PTC, mixed PTC carcinomas, history of thyroid surgery, prior head and neck radiation exposure, and distant metastasis. Ultimately, a total of 250 patients who underwent total thyroidectomy (TT) and bilateral CLND were enrolled in this study.

Before surgery, all patients underwent preoperative examinations, including physical examinations, high-quality ultrasonography (US) of the thyroid and cervical lymph nodes, and thyroid function testing. In this study, clinical N0 (cN0) was defined as patients without clinical evidence of any lymph node metastases on preoperative or intraoperative examination, and clinical N1 (cN1) was defined as patients with clinically evident lymph nodes based on preoperative physical examination, preoperative imaging evaluation, or intraoperative evidence of detectable lymph nodes according to the American Thyroid Association (ATA, 2015 Edition) [20]. Therapeutic CLND was performed when suspicious central LN metastasis was discovered during preoperative or intraoperative examination, and prophylactic CLND (ipsilateral or bilateral) was performed for these advanced PTC patients (T3/T4) or clinically involved lateral neck lymph nodes (cN1b); or this information is helpful for follow-up therapy [20].

### 2.2. Clinicopathological Variables Assessed

The clinicopathological variables, including age at diagnosis, sex, preoperative serum levels of thyrotropin (TSH), Hashimoto's thyroiditis (HT), maximal tumor size, multifocality, location, capsular invasion, extra thyroid extension (ETE), ipsi-CLNM, and ipsilateral lateral lymph node metastasis (ipsi-LLNM), as well as preoperative sonographic characteristics, including composition, echogenicity, margin, shape, microcalcifications, aspect ratio, and vascularity, were assessed. In addition, the BRAFV600E mutation status was assessed after surgery in all patients. In this study, the final histological diagnosis of primary tumors and cervical LNM was based on the final pathology report, and lateral neck nodes were divided into neck levels (II to V) based on the definition of the American Head and Neck Society (AHNS) [13].

### 2.3. Statistical Analysis

Statistical analyses were performed using SPSS 21.0 (IBM SPSS, Inc., Chicago, IL, USA) and R version 3.6.1. Continuous variables are presented as the mean ± standard deviation (SD), and categorical variables are presented as number, percentage (%), and odds ratio (OR) with confidence interval (CI). Variables with significance in univariate analysis were included in the multivariate logistic regression analysis to identify independent risk factors. Based on the identified risk factors, a nomogram of predictors associated with con-CLNM in uni-PTC patients was established using the program R. The area under the receiver operating characteristic curve (AUC), also known as the concordance index (C-index), was used to assess the discrimination ability of our nomogram. Finally, calibration plot and decision curve analysis (DCA) were performed to estimate the predicted probabilities and actual application value of the model, respectively. All tests were two-sided, and a *p*-value of <0.05 was considered statistically significant.

## 3. Results

### 3.1. Characteristics and Baseline of the Participants

A total of 250 patients with uni-PTC were enrolled, including 201 females (80.4%) and 49 males (19.6%), with a female-to-male ratio of 4.1 : 1. We divided the patients into two groups based on age using 55-year-old cutoff values according to the 8th American Joint Committee on Cancer (AJCC) staging systems [24]. The age range was 18–70 years (39.67 ± 11.36 years). The mean maximum diameter of the primary tumor was 15.95 ± 10.25 mm, with sizes ranging from 2 to 55 mm. In this study, a total of 38% of the study population had papillary microcarcinomas. Hashimoto's thyroiditis (HT) was found in 120 patients (48.0%). Other characteristics were BRAFV600E mutation in 179 (71.6%), multifocal lesions in 21 (8.4%), capsular invasion in 83 (33.2%), and ETE in 58 (23.2%) (see Table 1).

In this study, we recruited 130 cN0 patients and 120 cN1 patients and found 157 (62.8%, 68 in cN0 and 89 in cN1) patients with pathologically confirmed central lymph node metastasis (CLNM). Out of the 157 patients with CLNM, contralateral central lymph node metastasis (con-CLNM) was found in 75 (47.8%, 18 in cN0 and 57 in cN1) patients—including 72 patients with bilateral central lymph node metastasis (bi-CLNM) and 3 patients with skip-CLNM—only 82 patients (52.22%, 50 in cN0 and 32 in cN1) with ipsi-CLNM, and no con-CLNM. In addition, the numbers of con-CLNM and ipsi-CLNM patients were 2.99 ± 2.54 (range, 1–15) and 4.12 ± 3.30 (range, 1–20). In this study, 86 (34.40%) patients were observed to have ipsilateral lateral lymph node metastasis (ipsi-LLNM), of which 48 (55.81%) patients with con-CLNM (see Table 1).

### 3.2. Factors Influencing Con-CLNM in Patients with Uni-PTC Analyzed Using Logistic Regression

In the univariate analysis, age (*p* < 0.001), sex (*p* < 0.001), Hashimoto's thyroiditis (*p* < 0.01), sonographic characteristics (*p* < 0.05), capsular invasion (*p* < 0.001), ETE (*p* < 0.01), tumor size (*p* < 0.01), and pathologically confirmed ipsi-CLNM (*p* < 0.001) and ipsi-LLNM (*p* < 0.001) were closely related to con-CLNM (see Table 2).

The results of preoperative sonographic characteristics are listed in Table 2. In this study, hypoechogenic (*p* < 0.05) and microcalcifications (*p* < 0.01) showed significance between the con-CLNM (+) group and the con-CLNM (−) group, while solid composition, irregular shape, poor margins, and vascularity did not. The effects of Hashimoto's thyroiditis (HT) on the diagnosis of suspicious lymph nodes in the central compartment during preoperative ultrasound (US) are well proved [5]. Similarly, we found that HT significantly affected the diagnostic value of neck US for CLNM in uni-PTC patients, and the sensitivity, specificity, positive predictive value (PPV), and accuracy of neck US for CLNM were decreased in uni-PTC patients with HT (see Table 3).

**Figure 1:**
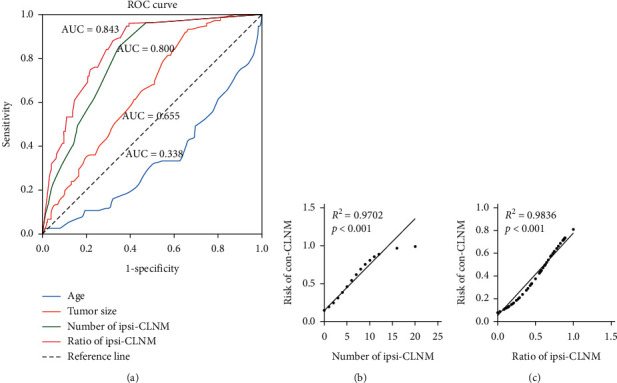
Diagnostic value of risk factors for predicting con-CLNM in uni-PTC patients. (a) ROC curve for age (blue line), tumor size (orange line), number of ipsi-CLNM (green line), and the ratio of ipsi-CLNM (red line). (b), (c) The correlation analyses of the number of ipsi-CLNM and risk of con-CLNM or the ratio of ipsi-CLNM and risk of con-CLNM in uni-PTC patients are shown, Pearson's r test, *n* = 250, *p* < 0.001. Con-CLNM, contralateral central lymph node metastasis; ipsi-CLNM, ipsilateral central lymph node metastasis; ROC curve, receiver operating characteristic curve; uni-PTC, unilateral papillary thyroid carcinoma.

Pathologically confirmed ipsi-CLNM or ipsi-LLNM was found to be related to con-CLNM. Interestingly, we found that patients with ipsi-CLNM or ipsi-LLNM were more likely to have con-CLNM (both *p* < 0.001); in addition, we found that both the number and ratio of ipsi-CLNM were positively correlated with con-CLNM (both *p* < 0.001) (see Table 2). In receiver operating characteristic (ROC) curve analysis, we found that the number of ipsi-CLNM and the ratio of ipsi-CLNM would be good predictors of con-CLNM; however, the accuracy of age and tumor size in diagnosing con-CLNM was limited. The optimal cutoff number of ipsi-CLNM in the con-CLNM (+) group was 1.5 (area under the curve (AUC) = 0.800, 0.744–0.855), and the optimal cutoff ratio of ipsi-CLNM in the con-CLNM (+) group was 0.16 (area under curve (AUC) = 0.843, 0.792–0.893) (see Figure 1(a)). In addition, we found that there was a significant correlation between the number of ipsi-CLNM or the ratio of ipsi-CLNM and the risk of con-CLNM (*R*^2^ = 0.9702, *p* < 0.001; *R*^2^ = 0.9836, *p* < 0.001, respectively) (see Figures 1(b) and 1(c)).

### 3.3. Contribution of the Factors to Con-CLNM in Uni-PTCs

To define the predictors of con-CLNM in uni-PTCs, we performed binary logistic regression analyses with sonographic and clinicopathological characteristics (see Table 4). Single-factor variables with *p* < 0.05, such as male sex, Hashimoto's thyroiditis (HT), tumor size ≥8.55 mm, hypoechoic or microcalcification on neck US, capsular invasion, present ETE, number of ipsi-CLNM ≥1.5, ratio of ipsi-CLNM ≥0.16, and present ipsi-LLNM were included in the logistic regression model. We found that male sex, capsular invasion, the ratio of ipsi-CLNM ≥0.16, and present ipsi-LLNM were significant predictors of con-CLNM (OR = 2.797, *p*=0.019; OR = 2.538, *p*=0.012; OR = 26.588, *p* < 0.001; OR = 2.202, *p*=0.033, respectively). However, uni-PTC with HT was statistically significantly different (OR = 0.430, *p*=0.020) (see Table 4); in other words, this was a protective factor for con-CLNM in uni-PTC patients.

### 3.4. Establishment of the Predictive Model by Nomogram

**Figure 2:**
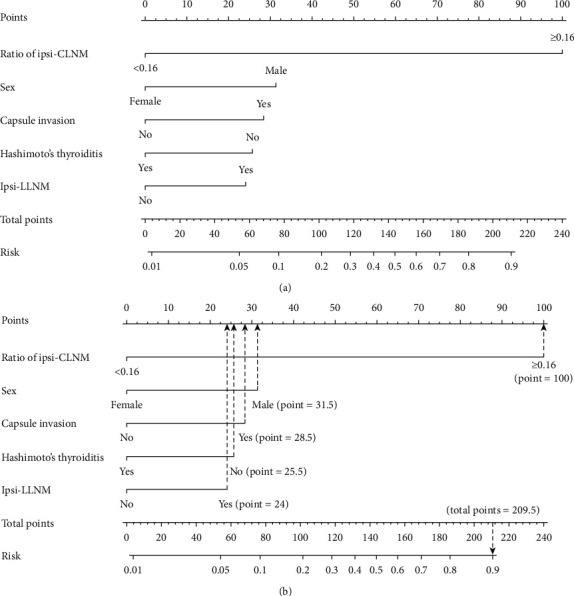
Generation of the con-CLNM score by nomogram. (a) Nomogram for predicting con-CLNM in uni-PTC patients. (b) A uni-PTC patient with the ratio of ipsi-CLNM ≥0.16, male sex, present capsular invasion, without Hashimoto's thyroiditis, and present ipsi-LLNM has a con-CLNM of 90%. Con-CLNM, contralateral central lymph node metastasis; ipsi-CLNM, ipsilateral central lymph node metastasis; ipsi-LLNM, ipsilateral lateral lymph node metastasis; uni-PTC, unilateral papillary thyroid carcinoma.

A nomogram that contained the significant factors related to con-CLNM was established based on the logistic regression model (see Figure 2(a)). In the nomogram, the risk level of each variable was assigned points in a score ranging from 0 to 100, and the ratio of ipsi-CLNM ≥0.16 was recognized as the largest contributor to the score (100) followed by male sex (31.5), present capsular invasion (28.5), without Hashimoto's thyroiditis (25.5), and present ipsi-LLNM (24). Then, the risk possibility of con-CLNM could be calculated for an individual patient. In our results, the probability of con-CLNM was determined by calculating the total score of independent risk factors if a patient with all of the above risk factors had an estimated 90% predicted probability of con-CLNM.  Figure 2(b) represents an example of how our nomogram could be used to predict the probability of con-CLNM in uni-PTC patients.

### 3.5. Clinical Utility of the Nomogram for Predicting Con-CLNM in Uni-PTCs

**Figure 3:**
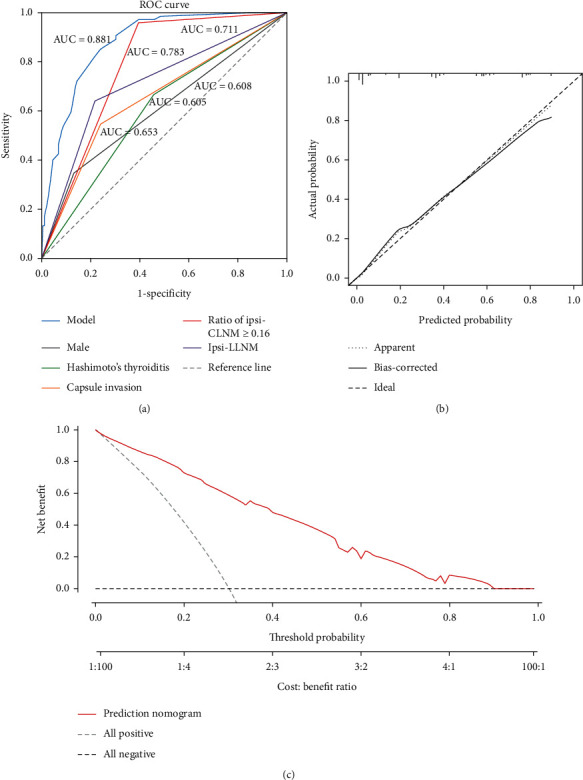
Predictive accuracy of the nomogram to predict con-CLNM in uni-PTC patients. (a) ROC curve for the model (blue line), male sex (gray line), Hashimoto's thyroiditis (green line), capsular invasion (orange line), the ratio of ipsi-CLNM ≥0.16 (red line), and ipsi-LLNM (purple line). (b) Calibration plots of the nomogram for predicting con-CLNM in uni-PTC patients. (c) Decision curve for nomogram to predict con-CLNM in uni-PTC patients. Con-CLNM, contralateral central lymph node metastasis; ipsi-CLNM, ipsilateral central lymph node metastasis; ipsi-LLNM, ipsilateral lateral lymph node metastasis; ROC curve, receiver operating characteristic curve; uni-PTC, unilateral papillary thyroid carcinoma.

To determine the predictive ability of the model, the area under the receiver operating characteristic curve (AUC), also known as the concordance index (C-index), was assessed. The predictive accuracy of the prediction model is shown in Figure 3(a). The model showed an AUC of 0.881 (95% CI 0.840–0.923) with a boundary value of 0.349, a Youden index of 0.613, a sensitivity of 85.3%, a specificity of 76.0%, a positive predictive value of 60.3%, and a negative predictive value of 92.4%. To clarify the applicable patient population of the nomogram, ROC curve was performed in cN0 and cN1 patients. The AUC in cN0 and cN1 was 0.897 and 0.895, respectively, which means the present nomogram is applicable in both cN0 and cN1 patients (see Supplementary Figure 1(a) and 1(b)). The calibration plot demonstrated strong agreement between predicted probability and actual risk of con-CLNM in the whole cohort (Hosmer–Lemeshow test, *p*=0.963) (see Figure 3(b)). Decision curve analysis (DCA) was employed to assess the improvement the prediction model brings to decision-making. Based on the threshold probability, we evaluated the actual application value of our prediction model through the decision curve analysis. In our results, when the prediction probability of con-CLNM in uni-PTC patients calculated by the nomogram was less than 0.3 or more than 0.9, actual application values based on the nomogram would be nonbenefit. Conversely, the model was useful when threshold probabilities were in the range between 0.3 and 0.9 (see Figure 3(c)).

## 4. Discussion

To our knowledge, this is the first study to evaluate both clinicopathological features and genetic background to predict con-CLNM in uni-PTC patients. We identified five risk factors significantly related to con-CLNM of uni-PTC including male sex, without HT, present capsular invasion, the ratio of ipsi-CLNM ≥0.16, and with ipsi-LLNM. Since few studies have attempted to develop predictive models for con-CLNM, the current ability to diagnose con-CLNM of uni-PTC is limited. In the study by Wei et al., four risk factors including primary capsular/extracapsular invasion, tumor size >1 cm, pretracheal/prelaryngeal LN metastasis, and lateral neck LN metastasis were related to contralateral paratracheal LN metastasis in uni-PTC, which was consistent with our results [25]. Regardless, our study developed a nomogram as a novel method to individually quantify the probability of con-CLNM in uni-PTC patients.

In our study, the incidence of CLNM was 62.8% (157/250), which was consistent with previous studies in which the incidence of CLNM was 21%–82% in PTC [7]. In addition, the incidence of con-CLNM was 30.0% (75/250), but 62.8% (54/86) presented in con-cN1 and 26.5% (18/68) presented in con-cN0 of PTC with ipsi-CLNM, which was consistent with previous studies that showed a 20–25% probability of con-CLNM in PTC with cN0 [15, 16]. Although previous studies have reported that the probability of con-CLNM was 34.3% in patients with ipsi-LLNM (ipsilateral pN1b) [26], the probabilities of con-CLNM were 55.8% (48/86) in our study and 32.9% (25/76) in PTC patients with ipsi-CLNM but absent ipsi-LLNM (pN1a). Because aggressive dissection in these patients will increase the risk of complications such as recurrent laryngeal nerve injury, therapeutic rather than prophylactic management of the contralateral central neck compartment will possibly benefit patients with uni-PTC.

Moreover, we also found that male sex, capsular invasion, and ipsi-LLNM increased the risk factors for con-CLNM, consistent with the previous studies [15–17]. However, in contrast with previous reports [17, 27], we did not find that primary tumor size was the independent risk factor for con-CLNM. Interestingly, we found that the ratio of ipsi-CLNM ≥0.16 in uni-PTC patients had a higher possibility for con-CLNM, and the number and ratio of ipsi-CLNM were positively correlated with the risk of con-CLNM. Although Hashimoto's thyroiditis (HT) increased the risk of PTC, several previous studies confirmed that preoperative imaging examinations, including US, CT, and MRI, showed poor accuracy of CLNM in PTC patients with HT, owing to abnormal lymphadenopathy in the central compartment caused by HT itself [28, 29]. Our previous study found that HT seems to be a protective factor of LNM in PTC patients [30, 31]. In this study, we found that the rate of con-CLNM was lower in uni-PTC with Hashimoto's thyroiditis than in those patients without Hashimoto's thyroiditis. Out of patients with evidence of con-CLNM in preoperative ultrasound, the incidence of pathologically confirmed con-CLNM was significantly higher in patients without Hashimoto's thyroiditis than in patients with Hashimoto's thyroiditis (71.4% vs. 32.6%); thus, whether Hashimoto's thyroiditis affects the lymph node metastasis of PTC through some inflammatory immune pathway needs further investigations.

While upstaging of patients due to the detection of lymph node metastasis status may change treatment, the impact of prognosis in PTC patients is most often variable. PTC is currently considered to be a low-risk malignancy with a good prognosis worldwide; however, CLNM and postoperative recurrence can reduce the disease-free survival (DFS) in patients with PTC [21, 32, 33]. Studies have confirmed that there are 20%–25% probabilities that PTC can occur with con-CLNM [15, 17]; bilateral CLND in advanced PTC has shown an improved DFS rate and reduced risk of recurrence [34, 35], though controversy exists in whether contralateral prophylactic CLND should be carried out in uni-PTC and advanced PTC [13, 14]. The incidence of residual nodal metastases in the contralateral central neck compartment was 14.3%–23.0% in PTC patients who underwent ipsilateral CLND, which was significantly higher than in those who underwent bilateral CLND [18]. Moreover, persistent or recurrent PTC caused by residues from initial surgery largely showed resistance to radioiodine treatment [36] and increased the risk of reoperation, hypocalcemia, and recurrent laryngeal nerve injury [37]. Therefore, the surgeon should carefully evaluate the probability of con-CLNM in uni-PTC patients based on preoperative clinical features, considering either the residue of occult con-CLNM in unilateral CLND or the increased chance of postoperative complications of bilateral CLND. According to our study results, bilateral CLND should be considered in those uni-PTC patients (male sex, capsular invasion, without HT, the ratio of ipsi-CLNM ≥0.16, and ipsi-LLNM), as a high risk of con-CLNM exists.

There are, however, some potential limitations to our study, including the inherent design defects due to its design as a retrospective study. In this study, the risk factors for con-CLNM were revealed, and the performance of internal validations proved the obvious discrimination ability of the prediction model; external validation should be added in a future study. Although contralateral central neck compartment LN status was evaluated, we were still unable to define whether prophylactic bilateral CLND could improve PTC patient outcome; thus, further investigations in enlarged, multicenter cohorts are needed. Furthermore, the 250 patients in this study are all Chinese, and whether these identified predictors also apply to other races requires more research evidence. Despite these limitations, the main advantage of our study is that we have identified the risk factors of con-CLNM and established a prediction model in uni-PTC patients.

## 5. Conclusion

In summary, we have identified several independent risk factors for con-CLNM in uni-PTC. The model based on predictive factors provided a novel way to predict quantitatively and accurately whether con-CLNM occurred in patients with uni-PTC. Based on the quantified risk stratification system proposed by the nomogram, uni-PTC patients with male sex, without HT, present capsular invasion, a ratio of ipsi-CLNM ≥0.16, and with ipsi-LLNM can be considered having a high risk of con-CLNM, and tailored therapeutic LND should be performed.

## Figures and Tables

**Table 1:** Demographics and clinicopathological characteristics of 250 unilateral papillary thyroid carcinoma patients.

Characteristics	Number (%)
No. of patients	250 (100)
*Sex*	
Male/female	49 (19.6)/201 (80.4)
Age (mean ± SD, (range)), years	39.67 ± 11.36 (18–70)
≥55/<55	25 (10.0)/225 (90.0)
Primary tumor size (mean ± SD), mm	15.95 ± 10.25
≤10/>10	95 (38.0)/155 (62.0)
*Hashimoto's thyroiditis*	
Yes/No	120 (48.0)/130 (52.0)
*BRAF* ^*V600E*^ *mutation*	
Positive/negative	179 (71.6)/71 (28.4)
*Multifocality*	
Yes/No	21 (8.4)/229 (91.6)
*Capsular invasion*	
Present/absent	83 (33.2)/167 (66.8)
*ETE*	
Present/absent	58 (23.2)/192 (76.8)
cN0/cN1	130 (52.0)/120 (48.0)
CLNM	157 (62.8)
Bi-CLNM	72 (45.9)
Only ipsi-CLNM	82 (52.2)
Skip-CLNM	3 (1.9)
*Number of CLNM* (*mean* *±* *SD, range*)	
Con-CLNM	2.99 ± 2.54 (1–15)
Ipsi-CLNM	4.12 ± 3.30 (1–20)
Ipsi-LLNM	86 (34.4)
With con-CLNM	48 (55.8)

Abbreviations: bi-CLNM, bilateral central lymph node metastasis; CLNM, central lymph node metastasis; cN0, clinical N0; cN1, clinical N1; con-CLNM, contralateral central lymph node metastasis; ETE, extrathyroid extension; ipsi-CLNM, ipsilateral central lymph node metastasis; ipsi-LLNM, ipsilateral lateral lymph node metastasis; PTC, papillary thyroid carcinoma; SD, standard deviation; Skip-CLNM, contralateral skip central lymph node metastasis.

**Table 2:** Univariate analysis of clinicopathological characteristics correlated with con-CLNM in uni-PTC.

Characteristics	Total (*n* = 250) (%)	Con-CLNM (+) (*n* = 75) (%)	Con-CLNM (−) (*n* = 175) (%)	*p*
Age (yrs)	39.67 ± 11.36	35.44 ± 11.38	41.49 ± 10.89	**<0.001** ^**b**^
Age ˂55	225 (90.0)	71 (94.7)	154 (88.0)	0.107^a^
Male sex	49 (19.6)	26 (34.7)	23 (13.1)	**<0.001** ^**a**^
TSH level	2.77 ± 4.21	2.44 ± 2.07	2.91 ± 4.84	0.420^b^
Hashimoto's thyroiditis	120 (48.0)	25 (33.3)	95 (54.3)	**0.002** ^**a**^
BRAF^V600E^ mutation	179 (71.6)	54 (72.0)	125 (71.4)	0.927^a^
Sonographic characteristics				
Solid composition	240 (96.0)	69 (92.0)	171 (97.7)	0.078^c^
Hypoechogenic	220 (88.0)	61 (81.3)	159 (90.9)	**0.034** ^**a**^
Irregular shape	182 (72.8)	58 (77.3)	124 (70.9)	0.292^a^
Poorly marginal	167 (66.8)	46 (61.3)	121 (69.1)	0.230^a^
Oval	50 (20.0)	12 (16.0)	38 (21.7)	0.301^a^
Microcalcifications	212 (84.8)	71 (94.7)	141 (80.6)	**0.004** ^**a**^
Vascularity				
Absent	58 (23.2)	17 (22.7)	41 (23.4)	0.381^a^
Few	120 (48.0)	32 (42.7)	88 (50.3)	
Abundant	72 (28.8)	26 (34.6)	46 (26.3)	
Pathological characteristics				
Multifocality	21 (8.4)	6 (8.0)	15 (8.6)	0.881^a^
Location of primary tumor				
Superior lobe	54 (21.6)	19 (25.3)	35 (20.0)	0.539^a^
Middle lobe	141 (56.4)	42 (56.0)	99 (56.6)	
Inferior lobe	55 (22.0)	14 (18.7)	41 (23.4)	
Capsular invasion	83 (33.2)	41 (54.7)	42 (24.0)	**<0.001** ^**a**^
ETE	58 (23.2)	28 (37.3)	30 (17.1)	**<0.001** ^**a**^
Tumor size (mm)	15.95 ± 10.25	19.30 ± 10.83	14.51 ± 9.68	**<0.001** ^**b**^
Diameter >10	155 (62.0)	59 (78.7)	96 (54.9)	**<0.001** ^**a**^
Pathologically confirmed ipsi-CLNM				
Present	154 (61.6)	72 (96.0)	82 (46.9)	**<0.001** ^**a**^
Ipsilateral central LN				
Metastasis number	2.54 ± 3.27	4.69 ± 3.88	1.61 ± 2.46	**<0.001** ^**b**^
Harvested number	7.62 ± 4.75	7.43 ± 4.64	7.70 ± 4.81	0.674^b^
Metastasis ratio	0.34 ± 0.35	0.64 ± 0.30	0.21 ± 0.29	**<0.001** ^**b**^
Pathologically confirmed ipsi-LLNM				
Present	86 (34.4)	48 (64.0)	38 (21.7)	**<0.001** ^**a**^

Note: Variables with statistical significance are shown in bold. ^a^Chi-square test, ^b^Student's *t* test, and ^c^Fisher's exact test were adopted. Abbreviations: con-CLNM, contralateral central lymph node metastasis; ETE, extrathyroid extension; ipsi-CLNM, ipsilateral central lymph node metastasis; oval, taller than wide; TSH, thyrotropin; uni-PTC, unilateral papillary thyroid carcinoma.

**Table 3:** Diagnostic value for suspicious lymph nodes in the contralateral central neck compartment of uni-PTC patients with or without Hashimoto's thyroiditis on neck US.

Parameter	Without Hashimoto's thyroiditis			Hashimoto's thyroiditis		
	Con-CLNM (+) (%)	Con-CLNM (−) (%)	*p*	Con-CLNM (+) (%)	Con-CLNM (−) (%)	*p*
Abnormal US	30 (71.4)	12 (28.6)	<0.001	14 (32.6)	29 (67.4)	0.033
Normal US	20 (22.7)	68 (77.3)		11 (14.3)	66 (85.7)	
Value of neck US in diagnosis of suspicious lymph nodes						
Sensitivity	60.0%			56.0%		
Specificity	85.0%			69.5%		
PPV	71.4%			32.6%		
NPV	77.3%			85.7%		
Accuracy	73.1%			66.7%		

Note: Variables with statistical significance are shown in bold; the chi-square test was adopted. Abbreviations: con-CLNM, contralateral central lymph node metastasis; uni-PTC, unilateral papillary thyroid carcinoma; PPV, positive predictive value; NPV, negative predictive value; US, ultrasonography.

**Table 4:** Binary logistic regression analysis for con-CLNM in uni-PTC.

Variables	*β*	OR	95% CI	*p*
Male sex	1.029	2.797	1.182–6.617	**0.019**
With HT	−0.844	0.430	0.211–0.876	**0.020**
Tumor size ≥8.55 mm	0.826	2.284	0.689–7.570	0.177
Hypoechoic on neck US	0.512	1.668	0.543–5.125	0.372
Microcalcifications on neck US	0.060	1.062	0.242–4.656	0.936
With capsular invasion	0.931	2.538	1.223–5.267	**0.012**
Present ETE	1.053	2.866	0.758–10.836	0.121
Number of ipsi-CLNM ≥1.5	0.550	1.732	0.580–5.177	0.325
Ratio of ipsi-CLNM ≥0.16	3.280	26.588	7.596–93.069	**<0.001**
Present ipsi-LLNM	0.789	2.202	1.064–4.557	**0.033**
Constant	−3.875	0.021	—	<0.001

Note: Variables with statistical significance are shown in bold. Abbreviations: con-CLNM, contralateral central lymph node metastases; ETE, extrathyroid extension; HT, Hashimoto's thyroiditis; ipsi-CLNM, ipsilateral central lymph node metastasis; ipsi-LLNM, ipsilateral lateral lymph node metastasis; OR, odds ratio; uni-PTC, unilateral papillary thyroid carcinoma; 95% CI, 95% confidence interval; *β*, regression coefficient.

## Data Availability

The data used to support the results of this study are included within this article.

## Supplementary Materials

Supplementary Materials Supplementary Figure 1: verification of the nomogram in cN0 and cN1 patients. (a) ROC curve for the present nomogram model (blue line) in cN0 patients. (b) ROC curve for the present nomogram model (blue line) in cN1 patients .
