# Supplementary material for: A Prediction Model for Contralateral Central Neck Lymph Node Metastases in Unilateral Papillary Thyroid Cancer
Source: Int J Endocrinol. 2021 Jun 30;2021:6621067. doi: 10.1155/2021/6621067 (PMC8263281; doi:10.1155/2021/6621067)
Supplement: Supplementary Materials — Supplementary Figure 1: verification of the nomogram in cN0 and cN1 patients. (a) ROC curve for the present nomogram model (blue line) in cN0 patients. (b) ROC curve for the present nomogram model (blue line) in cN1 patients . [file 6621067.f1.doc]

# Supplemental material

# A prediction model for contralateral central neck lymph node metastases in unilateral papillary thyroid cancer

Hai-long Tan^1^, Bo-qiang Huang^1^, Gui-you Li^1^, Bo Wei^1^, Pei Chen^1^, Hui-Yu Hu^1^, Mian Liu^1^, Deng-jie Ou-yang^1^, Qiong Yang^1^, Zi-en Qin^1^, Qi-man Shi^1^, Ning Li^1^, Peng Huang^1^, and Shi Chang^1, 2^

**Figure 1**


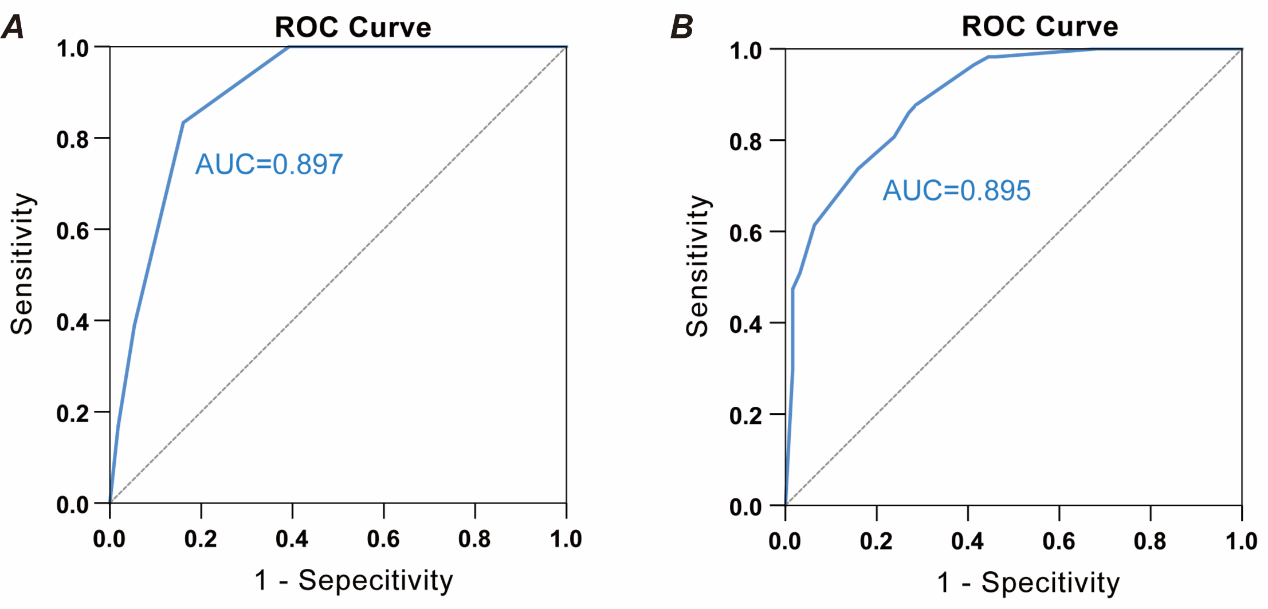


Figure 1. verification of the nomogram in cN0 and cN1 patients. (A) ROC curve for the present nomogram model (blue line) in cN0 patients. (B) ROC curve for the present nomogram model (blue line) in cN1 patients.
